# Lasting effects of residential mobility during childhood on psychopathology among Chinese University students

**DOI:** 10.1186/s12888-020-03018-9

**Published:** 2021-01-15

**Authors:** Yingzhe Zhang, Jeremy Coid, Xiang Liu, Yamin Zhang, Huan Sun, Xiaojing Li, Wanjie Tang, Qiang Wang, Wei Deng, Liansheng Zhao, Xiaohong Ma, Yajing Meng, Mingli Li, Huiyao Wang, Ting Chen, Qiuyue Lv, Wanjun Guo, Tao Li

**Affiliations:** 1 Mental Health Center and Psychiatric Laboratory, the State Key Laboratory of Biotherapy, West China Hospital of Sichuan University, No. 28 Dianxin South street, Chengdu, 610041 Sichuan China; 2 West China Brain Research Center, West China Hospital of Sichuan University, Chengdu, Sichuan China; 3 Mental Health Education Center, Sichuan University, Chengdu, Sichuan China; 4 West China School of Public Health and West China Fourth Hospital, Sichuan University, Chengdu, Sichuan China; 5 Centre for Psychology Education and Consultation, Sichuan University, Chengdu, China

**Keywords:** Residential mobility, Childhood maltreatment, Psychiatric morbidity, Student mental health, Developmental timing

## Abstract

**Background:**

Residential mobility during childhood increases risk of psychopathology in adulthood and is a common experience among Chinese children. This study investigated associations between number and age of first move, etiological risk factors for psychopathology, and common mental disorders in adolescence and early adulthood.

**Methods:**

The sample included 39,531 undergraduates (84.5% completion rate) age 15–34 years in their first year at a Chinese comprehensive university in annual cross-sectional surveys during 2014–2018. Common mental disorders measured using standardised self-report instruments. Data analysed using logistic regression models and interaction analysis.

**Results:**

Half of all students experienced one or more moves of residence before age 15 years. Outcomes of Depression, Somatisation, Obsessive-compulsive disorder, Hallucinations and Delusions, and Suicide attempts showed dose-response relationships with increasing number of moves. Other etiological risk factors, including childhood disadvantage and maltreatment, showed similar dose response relationships but did not confound associations with mobility. We found interactions between reporting any move and being a left-behind child on depression and somatisation; number of moves and younger age at first move on depression, somatisation, suicide attempts and hallucinations and delusions.

**Conclusions:**

Residential mobility in childhood is associated with psychopathology in adulthood and this association increases with increasing number of moves. Mobility is also associated with childhood disadvantage and maltreatment but associations with psychopathology are independent of these factors. Multiplicative effects were shown for multiple moves starting at a younger age and if the participant had been a left-behind child.

## Background

The People’s Republic of China has undergone the largest and most rapid process of urbanization of any country from the mid-twentieth century onwards. Persons recorded as living in urban areas increased from 13 to 57% between 1952 and 2016, with rural to urban labour migration providing the backbone for long-term economic transformation. A substantial proportion of children in China either move with their parents or are left behind in the countryside. The effects of being left behind by parents on children’s psychological development and the risk of subsequent adverse effects on psychiatric morbidity in adulthood are increasingly recognized [1, 2]. However, effects of residential mobility during childhood have received little study in China despite approximately one third of children of rural to urban work migrants accompanying their parents, sometimes to unstable and temporary working conditions with poor living conditions and healthcare.

Previous research has suggested that the associations between residential moves in childhood and psychopathology are complex but can be divided into three main areas:

### Disrupted attachments and life events

MT Merrick, M Henly, HA Turner, C David-Ferdon, S Hamby, A Kacha-Ochana, TR Simon and D Finkelhor [3] described predictability in a child’s environment as a critical quality of safe, stable, nurturing relationships and environments which promote wellbeing and protects against maltreatment. Residential mobility can disrupt social networks, emotional ties, family routines, and schooling. It can be especially stressful for all family members if the move is not voluntary [4]. Moving house is considered a stressful life event [5], infants from age 7 months can appraise situations as stressful, and from 12 months use social referencing (cues from reactions of parents and siblings) to assess events and respond emotionally or behaviourally [6]. Stressful life events in general are a known risk factor for onset of major depressive disorder [7, 8]. However, the intensity of stress produced by life events is thought more important than the weight of life events themselves in evaluating the psychological impact of life events [9]. Furthermore, cumulative life events have greater impact and a significant adverse impact can occur when multiple events occur over a relatively short time period.

Moving home or school is usually associated with short-term social and health effects in children and not usually related to persistence into adulthood. Nevertheless, it has been claimed that a single school move, especially in primary grades, can be traumatic for a child and that signs of unresolved psychological pain, such as hitting, bullying, bragging, lying and sometimes withdrawal can be observed and where children’s reactions to moving may include fear of parental abandonment, depression and anger [10]. In young children, symptoms such as refusing to eat, being clingy, and becoming aggressive or shy are not uncommon. However, in older school-aged children, moves are more likely to result in changes in sleep patterns, having trouble concentrating, and suffering stomach or headaches [11].

### Child maltreatment

Studies in Europe and North America have found that children who move more frequently from birth until their mid-teens are more likely to experience child maltreatment [12, 13] and more likely to have subsequent histories of attempted suicide, violent criminality, mental illness and substance misuse, with higher rates of both natural and unnatural deaths when followed up into middle-age [14]. A second area of risk that may be of particular relevance to China is increased risk of child maltreatment during periods of residential instability through lack of parental supervision and protection from maltreatment by others when parents work long hours as migrant workers. Furthermore, child-parent conflict [15] can manifest as physical abuse from parents who are themselves stressed by their working conditions. YJ Gao, S Atkinson-Sheppard and X Liu [16] found parent-to-child abuse was more prevalent among migrant than local adolescents in Shenzhen city, with migrant adolescents more likely to be psychologically and physically abused by their parents than their local counterparts. Low academic performance, delinquent behaviour, family economic adversity, and low parent attachment put migrant adolescents at increased risk of both psychological and physical maltreatment.

### Family dysfunction

In western studies, important factors thought to contribute to risk of future psychiatric morbidity from residential mobility in childhood are serious and enduring difficulties within families rather than the direct effects of residential mobility. Frequent moves may be a proxy of family dysfunction and psychosocial adversity and occur more frequently among single parent and step-families and among those from lower socioeconomic backgrounds [17–19]. In many cases, moves may be due to debt and difficulty paying rent for accomodation. These family factors correspond to increased risk of behaviour-related disorders in adulthood [20–23]. Some families become residentially mobile because of family dysfunction and psychosocial adversity rather than moving for better economic opportunities, with concentration of dysfunctional families observed in inner urban areas characterised by socioeconomic deprivation [24]. These correspond in turn to consistently observed highest risks for behaviour-related disorders found in areas of concentrated deprivation such as antisocial personality disorder and substance misuse which are somewhat higher than common mental disorders, including anxiety and depression [25]. It may also provide an explanation for the greater impact of moves, particularly frequent moves, which occur during adolescence on psychopathology, and particularly on the behaviour-related disorders [14, 26, 27].

Family dysfunction, as conceptualised in western studies, occurs in China but has received little study. However, despite major social changes and increase in rate of divorce following rapid urbanisation [28], single and step-parenthood (other than following divorce and death) still remain uncommon in China. Furthermore, mass rural-to-urban migration in China is overwhelmingly for the purpose of increasing economic status aimed to improve family circumstances rather than the result of family dysfunction, albeit in the long-term and with uncertain outcome. Less is known about concentration of families with multiple problems in urban areas. Multiple problems associated with concentrated poverty are associated more with rural than urban living in China, although this could change in the future. Nevertheless, YJ Gao, S Atkinson-Sheppard and X Liu [16] found that factors of family economic adversity together with neighbourhood disorganisation in Shenzhen city increased risk of maltreatment.

### Residential mobility and psychiatric morbidity

Studies in USA, Europe and Australia which have investigated residential mobility by measuring moves of residence during childhood are consistent in showing associations with psychopathology as primary outcome over a range of different ages from 9 years until midlife. The more moves that have taken place the stronger these associations [4, 14, 19, 24, 26, 27, 29–32]. However, studies which have investigated the effects of moves beginning during critical time periods are less consistent. Externalizing disorders and psychosis are more likely following moves beginning in late childhood and adolescence. Other studies using generalised measures of psychopathology which have not discriminated between diagnostic categories suggest greater effects from moves beginning in early childhood [4, 14, 25, 26, 29, 31, 32]. None of these previous studies have used standardised research diagnostic instruments, except a single measure of anxiety in a Scottish study [24] and a major depressive episode in a US study [29]. Only one study [25] has investigated associations between residential moves and a full range of mental disorders using clinical diagnoses. This showed that most disorders were associated with childhood residential mobility rather than during adolescence, particularly externalizing disorders of antisocial personality disorder, substance misuse disorder (especially cannabis), and to a lesser extent psychotic disorder, including schizophrenia. Weaker associations were shown with anxiety disorders and depression.

### Current study

China has recognised the increasing importance of higher education for its future economy with massive expansion of capacity in universities. College students are therefore a key group in China in terms of human capital [33] because they will drive future economic growth and innovation as the country becomes less reliant on low cost labour for its economic development. However, first occurrence of several mental disorders coincides with age of college entry [34–41], particularly mood, anxiety, and substance use disorders. Furthermore, meta-analysis of international studies reported that an average of 30.6% of students suffer from depression, suggesting they are at higher risk than the general population [38]. These findings mean that prevention as well as intervention are important and indicate the need for better understanding of the mechanisms whereby key factors such as residential mobility lead to adverse effects on mental health in later life.

Because common mental disorders including depression and anxiety disorders affect a considerably larger proportion of the population, and because psychotic experiences (PEs) are more common than major psychotic illnesses such as schizophrenia and thought to be on a continuum with schizophrenia in the general population [42], we carried out a study of associations between residential mobility and these common conditions. In the context of China, the majority of moves involving children will have been planned by their parents with the long-term aim of better financial security and avoidance of rural poverty. When studying an undergraduate sample, it is therefore likely that a substantial proportion will have benefitted by early adulthood from their parents moving to improve their family’s financial circumstances during childhood, which can later help support them financially at university. Nevertheless, many undergraduates in China may have experienced disrupted attachments and adverse life events related to earlier residential instability. They may also have been at increased risk of maltreatment resulting in increased risk of mental disorder in early adulthood.

We are not aware of a previous study of effects of childhood residential mobility on students’ mental health in China. This study investigates associations between residential mobility during childhood and psychopathology in adulthood in a large sample of Chinese undergraduates. Our aims were to investigate (1) the associations between frequency of moves during childhood, age at first move, and psychopathology in adulthood, (2) whether additional etiological risk factors for adult psychopathology (including childhood maltreatment/disadvantage) are more prevalent among participants who moved during childhood and whether these factors confound associations between mobility and adult psychopathology, and (3) whether there is effect measure modification by age at first move and additional etiological risk factors on the associations observed between moves during childhood and psychopathology.

## Methods

### Participants

The Sichuan University Students Study is an on-going investigation into mental health problems associated with student life, risk factors preceding university entry, and their impact on academic performance and mental health. All freshmen admitted to the university are invited annually to complete a questionnaire on-line with a follow-up subsample at 1 year. The first year cross-sectional study sample in which students were asked to participate 1–3 months after university entry was used for this investigation and included 46,774 respondents, 2014–2018. After excluding those who gave incomplete information and under 15 years old, 39,531 were included, an 84.5% completion rate.

The study was approved by the Medical Ethics Committee of West China Hospital of Sichuan University.

### Measurement

#### Moves of residence

All participants were asked to report their birthplace, the number of moves of home (implying residence) they experienced from birth to 15 years, and age at home moves. In this survey, we adopted a five-level rating method used by PB Mortensen, CB Pedersen, T Westergaard, J Wohlfahrt, H Ewald, O Mors, PK Andersen and M Melbye [43], which ranks the residence place from 5 to 1 and which corresponds in China to geographical areas designated countryside, township, county-level city, prefecture-level city, and provincial capital/ administrative municipal city, respectively. For the purpose of rating rural birth, we combined levels 1 and 2 as rural areas.

Because it is reported that the upbringing environment during the first 15 years of life plays an essential role [44, 45], we only included moves of home before age 15. We categorized the number of moves before age 15 into 4 groups: never moved home (reference), moved once, moved twice, moved three times or more as categorical independent variables. In terms of the critical age periods at movements, we used the age at their first movement to indicate the exposure timing of residential instability, categorized into 3 periods: 0–5, 6–10 and 11–15 years.

#### Psychiatric morbidity

The PHQ-9 Depression module of the Prime-MD diagnostic instrument for common mental disorders [46] measured Depressive symptoms over the past 2 weeks, with a categorical measure of probable Depressive disorder at 15 and above, based on a previous meta-analysis [47].

The PHQ-15 module of Somatic symptoms provided a continuous measure over the past month with a cut-off of 10 rating a categorical diagnosis of Somatization [48]. The Obsessive-Compulsive Inventory-Revised (OCI-R) [49] provided a continuous measure of current beliefs and rituals with cut-off of 21 for probable Obsessive-Compulsive disorder. Participants additionally reported whether they had ever attempted suicide.

The Symptom Checklist-90-Revised (SCL-90-R) [50] assessed Psychotic experiences. Two symptom dimensions relevant to psychosis include 10 items in the psychoticism and 6 items in the paranoia subscales. Scores ranged from 0 “not at all” to 4 “extremely” on a 5-point Likert scale. To obtain a categorical psychosis measure, we re-coded SCL-R-90 items as symptoms, considered present when scoring 2 (moderate) or above for Hallucinations and Delusions, present when Hearing Voices and two of four persecutory items in the Paranoid Ideation sub-scale were rated ≥2.

#### Childhood maltreatment and adversity

Participants were asked if they had been physically or sexually abused, emotionally neglected, sexual abused or left behind (lived separated from parents more than 6 months) before 16 years old using the Childhood Section of the Chinese World Mental Health Initiative Composite International Diagnostic Interview [51, 52].

#### Demographics and putative etiological risk factors

Our analysis focuses primarily on the effect of childhood residential instability on the odds of psychiatric morbidity in adulthood, including depressive disorder, somatisation disorder, obsessive-compulsive disorder, hallucinations and delusions, and suicide attempts. However, we also investigated associations between moves and demographic factors as outcome (including age, sex, ethnic minority status, family income) to identify potential confounders.

We additionally investigated associations with a series of other putative risk factors for psychiatric morbidity. Participants were asked about their family’s current annual income. Low family income was defined as < 10,000 Yuan. They were asked if their family members had suffered from and received a diagnosis from a list of mental disorders. These were categorised into psychotic and non-psychotic illness among first degree relatives. They were classified according to whether they were rural born, as described above. They were asked about childhood adversity, including loss of parent through divorce or death (ever), and childhood maltreatment including physical and sexual abuse, emotional neglect before age 16 using the Chinese World Mental Health Initiative Composite International Diagnostic Interview [51, 52]. They were asked whether they had been a left-behind child, before age 16 years.

Our methodological strategy was to stratify number of moves of residence before 15 years with no moves of residence as our reference category and investigate associations with psychopathology. Because residential moves may be associated with other etiological factors which are independently associated with psychopathology, we investigated whether these etiological factors were sufficiently prevalent and associated with residential moves in our sample to be an additional or alternative explanation for psychiatric morbidity associated with increasing number of moves. We also identified potential demographic confounders associated with moves. We carried out three primary analyses: we firstly tested associations between age of first move and psychopathology. We next identified potential etiological confounders of the associations between residential moves and categories of psychiatric morbidity. Then we tested these associations before and after adjusting for these potential confounders. We hypothesised that younger age at first move and childhood adversity and maltreatment would moderate the effects of residential moves on psychopathology. We finally tested interactions between moves and these potential moderators.

### Statistical analysis

We initially investigated associations between the frequency of residential mobility and demography, etiological risk factors and common mental disorder. Multinomial logistic regression analyses were applied to explore the association between demography and etiological risk factors and frequency of moves (outcome). Binomial logistic regression analyses were applied to show the association between the frequency of moves and psychopathology (outcome).

To investigate the role of age of first move, we conducted stratification analyses and interaction analyses to test associations of age at first move and frequency of moving home on psychiatric disorders.

We finally tested associations between etiological variables and psychopathology for the following interaction analyses: move (any) and etiological variables, including rural birth, loss of parents, being a left-behind child, and childhood maltreatment, on psychopathology. In the interaction analyses, age of first move was reversely recoded.

All independent association analyses were performed in SPSS 25.

## Results

In total, 19,594 (49.6%) students reported they had experienced one or more moves of residence, with 29.9% reporting one, 13.5% reporting two, and 6.2% reporting three or more.

Table 1 shows the associations between move frequencies, demographics and etiological variables. Younger students were more likely to report residential mobility during childhood, with a trend for this association with younger age to increase with increasing number of moves. Female students and those with low family income were more likely to report a single move. A family history of psychosis (but not non-psychotic disorder) was associated with residential moves. Rural birth showed a more complex pattern of association with fewer students reporting a single or two moves but more reporting multiple moves. Childhood disadvantage and maltreatment, including reporting being a left behind child, physical abuse and emotional neglect showed a pattern of increasing association with increasing number of moves. Sexual abuse showed an association with 3 or more moves.

**Table 1:** Associations between number of Moves before 15 years and Demographic and Etiological Variables (*n*=39,531)

	No Moves (Ref.) *N*=19,937 (50.4%)	Moved 1 Time *N*=11,822 (29.9%)		Moved 2 Times *N*=5,344 (13.5%)		Moved 3 Times or more *N*=2,430 (6.2%)	
	Mean (SD) β (95%CI)	Mean (SD)	*β (*95%CI)	Mean (SD)	*β* (95%CI)	Mean (SD)	*β* (95%CI)
Demographics							
Age 18.71 (2.14%)	18.96 (2.39)	18.52 (1.91)	-0.44*** (-0.48- -0.38)	18.39 (1.63)	-0.28*** (-0.31- -0.26)	18.41 (1.80)	-0.18*** (-0.23- -0.13)
	N (%)	N (%)	OR (95%CI)	N (%)	OR (95%CI)	N (%)	OR (95%CI)
Male *n*=19,967 (50.5%)	10,259 (51.5)	5,815 (49.2)	0.91*** (0.87-0.96)	2,699 (50.5)	0.95 (0.90-1.00)	1,194 (49.1)	0.93 (0.81-1.07)
Ethnic Minority *n*=3,948(10.0%)	1,997 (10.0)	1,174 (9.9)	0.98 (0.91-1.06)	522 (9.8)	0.96 (0.87-1.07)	255 (10.5)	1.04(0.91-1.20)
Low Family Income *n*=6,351 (16.1%)	3,308 (16.6)	1,819 (15.4)	0.93* (0.87-0.99)	816 (15.3)	0.92 (0.85-1.01)	408 (16.8)	1.03 (0.92-1.16)
Family History							
Family History (psychosis) *n*=275(0.7%)	115 (0.6)	95 (0.8)	1.42* (1.08-1.87)	43 (0.8)	1.43* (1.01-2.03)	22 (0.9)	1.61* (1.02-2.55)
Family History (non-psychotic) n=306(0.8%)	144 (0.7)	89 (0.8)	1.03 (0.79-1.34)	49 (0.9)	1.25 (0.90-1.74)	24 (1.0)	1.35 (0.87-2.08)
Rural Birth							
Rural Birth *n*=21,307 (53.9%)	11,274 (56.6)	5,794 (49.0)	0.78*** (0.74-0.81)	2,765 (51.7)	0.88*** (0.83-0.93)	1,474 (60.7)	1.28*** (1.17-1.39)
Adverse Childhood Experiences							
Loss of Parent (Divorce) *N*=1,579 (4.0%)	805 (4.0)	466 (4.0)	0.97 (0.87-1.10)	196 (3.7)	0.90 (0.77-1.06)	112 (4.6)	1.15 (0.94-1.40)
Loss of Parent (Death) *N*=452 (1.1%)	219 (1.1)	135 (1.1)	0.99 (0.80-1.23)	59 (1.1)	0.95 (0.71-1.27)	39 (1.6)	1.38 (0.98-1.95)
Left behind (>6 months) *n*=9,463 (23.9%)	4,446 (22.3)	2,599 (22.0)	1.06* (1.01-1.12)	1,469 (27.5)	1.41*** (1.31-1.51)	949 (39.1)	2.22*** (2.03-2.43)
Childhood Maltreatment							
Physical Abused *N*=10,322 (26.1%)	4,872 (24.4)	3,104 (26.2)	1.14*** (1.08-1.20)	1,513 (28.3)	1.26*** (1.17-1.35)	833 (34.3)	1.68*** (1.53-1.84)
Sexual Abused *N*=1,146 (2.9%)	539 (2.7)	343 (2.9)	1.06 (0.92-1.21)	165 (3.1)	1.13 (0.95-1.35)	99 (4.1)	1.47*** (1.18-1.83)
Emotional Neglect *n*=13,365 (33.8%)	6,188 (31.0)	3,932 (33.3)	1.12*** (1.07-1.18)	2,077 (38.9)	1.43*** (1.34-1.52)	1,168 (48.1)	2.05*** (1.88-2.24)

Adjusted for age and sex, except for age and sex variables

*p< 0·05, **p< 0·01, ***p< 0·001

Table 2 shows associations between age at first move, number of moves and psychopathology. There was a trend for moves beginning at a younger age (0–5 years) to show higher odds of association with depression, suicide attempts, somatic disorder and obsessive-compulsive disorder than moves between 6 and 10 years. Significant associations with hallucinations and delusions were only observed if moves occurred between birth and 5 years. There were no associations between first moves occurring age 11–15 years and any category of psychopathology, except for suicide attempts when there had been 3 or more moves. Table 2 shows a general trend of increasing odds of association between first moves 0–5 and 6–10 years and psychopathology with increasing number of moves experienced during these age periods.

**Table 2:** Associations between Age at First Move and Psychopathology stratified by number of moves (*n*=39,531)

Psychopathology		Any move					
	No move	Moved 0-5y		Move 6-10y		Moved 11-15y	
	N (%)	N (%)	OR (95%CI)	N (%)	OR (95%CI)	N (%)	OR (95%CI)
Depression *N*=690(3.5%)	532(2.7)	251(4.3)	1.62*** (1.39-1.89)	276(3.4)	1.27*** (1.10-1.48)	162(2.8)	1.05 (0.88-1.26)
Suicide Attempt *N*=329(1.7%)	221(1.1)	120(2.1)	1.88*** (1.50-2.35)	131(1.6)	1.47*** (1.18-1.82)	78(1.4)	1.23 (0.95-1.60)
Somatic Disorder *N*=1374(7.0%)	1054(5.3)	463(8.0)	1.50*** (1.33-1.68)	565(7.0)	1.31*** (1.18-1.46)	344(6.1)	1.11 (0.98-1.26)
Obsessive Disorder *N*=822(4.2%)	602(3.0)	275(4.7)	1.51*** (1.30-1.74)	353(4.4)	1.40*** (1.23-1.60)	193(3.4)	1.09 (0.92-1.28)
Hallucinations and delusions *N*=173 (0.9%)	120(0.6)	74(1.3)	2.01*** (1.50-2.70)	59(0.7)	1.16 (0.85-1.59)	39(0.7)	1.10 (0.76-1.58)
		Moved 1 Time *N*=11,825 (29.9%)					
	No move	Moved 0-5y		Move 6-10y		Moved 11-15y	
	N (%)	N (%)	OR (95%CI)	N (%)	OR (95%CI)	N (%)	OR (95%CI)
Depression *N*=349(3.0%)	532(2.7)	75(3.2)	1.24(0.97-1.59)	135(2.9)	1.11(0.92-1.35)	139(2.9)	1.08(0.90-1.31)
Suicide Attempt *N*=166(1.4%)	221(1.1)	35(1.5)	1.35(0.94-1.93)	68(1.5)	1.32*(1.00-1.73)	63(1.3)	1.16(0.88-1.54)
Somatic Disorder *N*=752 (6.4%)	1054(5.3)	164(7.0)	1.32***(1.11-1.57)	306(6.6)	1.24***(1.09-1.42)	282(5.8)	1.08(0.94-1.24)
Obsessive Disorder *N*=445 (3.8%)	602(3.0)	95(4.0)	1.27*(1.02-1.59)	190(4.1)	1.32**(1.11-1.55)	160(3.3)	1.06(0.89-1.27)
Hallucinations and delusions *N*=93 (0.8%)	120(0.6)	24(1.0)	1.57(1.01-2.48)	36(0.8)	1.251(0.86-1.82)	33(0.7)	1.10(0.75-1.62)
		Moved 2 Times *N*=5,344 (13.5%)					
	No move	Moved 0-5y		Move 6-10y		Moved 11-15y	
	N (%)	N (%)	OR (95%CI)	N (%)	OR (95%CI)	N (%)	OR (95%CI)
Depression *N*=199 (3.7%)	532(2.7)	83(4.0)	1.55***(1.22-1.96)	98(3.9)	1.45***(1.17-1.81)	18(2.4)	0.87(0.54-1.41)
Suicide Attempt *N*=89 (1.7%)	221(1.1)	39(1.9)	1.71**(1.21-2.41)	39(1.5)	1.39(0.98-1.96)	11(1.5)	1.31(0.71-2.41)
Somatic Disorder *N*=391 (7.3%)	1054(5.3)	166(8.0)	1.52***(1.28-1.81)	171(6.8)	1.26**(1.06-1.49)	54(7.2)	1.32(0.99-1.80)
Obsessive Disorder *N*=226 (4.2%)	602(3.0)	93(4.5)	1.41**(1.13-1.77)	104(4.1)	1.31*(1.06-1.62)	29(3.9)	1.23(0.84-1.80)
Hallucinations and delusions *N*=43 (0.8%)	120(0.6)	23(1.1)	1.78*(1.14-2.80)	15(0.6)	0.95(0.55-1.63)	5(0.7)	1.07(0.43-2.61)
		Moved 3 Times or more *N*=2,430 (6.1%)					
	No move	Moved 0-5y		Move 6-10y		Moved 11-15y	
	N (%)	N (%)	OR (95%CI)	N (%)	OR (95%CI)	N (%)	OR (95%CI)
Depression *N*=142 (5.8%)	532(2.7)	94(6.6)	2.48***(1.97-3.11)	43(4.7)	1.71***(1.24-2.34)	5(5.3)	1.98(0.80-4.90)
Suicide Attempt *N*=74 (3.0%)	221(1.1)	46(3.3)	3.00***(2.17-4.15)	24(2.6)	2.41***(1.57-3.70)	4(4.3)	3.89**(1.41-10.69)
Somatic Disorder *N*=231 (9.5%)	1054(5.3)	135(9.5)	1.76***(1.46-2.13)	88(9.6)	1.82***(1.44-2.29)	8(8.5)	1.53(0.73-3.17)
Obsessive Disorder *N*=151 (6.2%)	602(3.0)	88(6.2)	2.01***(1.60-2.54)	59(6.4)	2.09***(1.59-2.76)	4(4.3)	1.33(0.49-3.63)
Hallucinations and delusions *N*=37 (1.5%)	120(0.6)	28(2.0)	3.16***(2.08-4.80)	8(0.9)	1.38(0.67-2.84)	1(1.1)	1.54(0.23-11.86)

Adjusted for sex, age, ethnic minority low family income, and rural birth

*P < .05, **P < .01, ***P < .001

Table 3 shows associations between psychopathology and putative etiological factors. There were no significant associations between family history of psychiatric disorder (both psychosis and non-psychosis) or with parental divorce. Rural birth and death of parents were both significantly associated with depression but not with other psychopathology. In contrast, associations with being a left behind child and childhood maltreatment were found for all categories of psychopathology.

**Table 3:** Associations between Etiological Variables and Psychopathology (*n*=39,531)

	Depression *N*=1,221 (3.1%)		Suicide Attempt *N*=550 (1.4%)		Somatic Disorder *N*=2,426 (6.1%)		Obsessive Disorder *N*=1,423 (3.6%)		Hallucinations and delusions *N*=292 (0.7%)	
Family History	N (%)	OR (95%CI)	N (%)	OR (95%CI)	N (%)	OR (95%CI)	N (%)	OR (95%CI)	N (%)	OR (95%CI)
Family History (psychosis) *n*=275 (0.7%)	6 (0.5)	0.71 (0.31-1.59)	6 (1.1)	1.61 (0.71-3.63)	13 (0.5)	0.78 (0.44-1.36)	7 (0.5)	0.70 (0.33-1.50)	1 (0.3)	\
Family History (non-psychotic) *n*=306(0.8%)	8 (0.7)	0.83 (0.41-1.68)	5 (0.9)	1.17 (0.48-2.83)	22 (0.9)	1.16 (0.75-1.79)	6 (0.4)	0.53 (0.23-1.18)	0 (0.0)	\
Left behind (>6 months) *n*=9,463 (23.9%)	460 (37.7)	1.98*** (1.76-2.23)	192 (34.9)	1.73*** (1.45-2.06)	763 (31.5)	1.52*** (1.39-1.66)	445 (31.3)	1.48*** (1.32-1.66)	88 (30.1)	1.39** (1.08-1.79)
Rural Birth										
Rural Birth *n*=21,307(53.9%)	766 (62.7)	1.51*** (1.34-1.70)	296 (53.8)	1.02 (0.86-1.21)	1,366 (56.3)	1.18 (1.08-1.28)	738 (51.9)	0.97 (0.87-1.08)	161 (55.1)	1.12 (0.89-1.41)
Loss of Parent (Divorce) *N*=1,579 (4.0%)	50 (4.1)	1.03 (0.77-1.37)	29 (5.3)	1.35 (0.92-1.97)	82 (3.4)	0.83 (0.67-1.05)	55 (3.9)	0.97 (0.73-1.27)	9 (3.1)	0.76 (0.39-1.49)
Loss of Parent (Death) *N*=452 (1.1%)	30 (2.5)	2.23*** (1.53-3.24)	6 (1.1)	0.94 (0.42-2.12)	36 (1.5)	1.29 (0.92-1.82)	10 (0.7)	0.58 (0.31-1.09)	4 (1.4)	\
Childhood Maltreatment										
Physical Abused *N*=10,322 (26.1%)	553 (45.3)	2.58*** (2.30-2.90)	277 (50.4)	3.15*** (2.66-3.74)	838 (34.5)	1.73*** (1.58-1.88)	525 (36.9)	1.73*** (1.55-1.94)	126 (43.2)	2.31*** (1.82-2.92)
Sexual Abused *N*=1,146 (2.9%)	86 (7.0)	2.53*** (2.01-3.18)	44 (8.0)	2.82*** (2.06-3.87)	124 (5.1)	1.69*** (1.40-2.05)	67 (4.7)	1.67*** (1.30-2.15)	30 (10.3)	3.72*** (2.53-5.46)
Emotional Neglect *n*=13,365 (33.8%)	758 (62.1)	3.38*** (3.01-3.81)	350 (63.6)	3.55*** (2.98-4.23)	1,204 (49.6)	2.09*** (1.92-2.27)	753 (52.9)	2.28*** (2.05-2.53)	184 (63.0)	3.41*** (2.68-4.33)

Cases < 5 were not estimated in models. Adjusted for age and sex

*p< 0·05, **p< 0·01, ***p< 0·001

Table 4 shows associations between number of moves before 15 years and psychopathology with adjustment for demographics in model 1 and with further adjustment for etiological risk factors found associated with psychopathology (see Table 3) in model 2. Associations with psychopathology showed increasing odds of association with increasing number of moves, for all categories, although a single move was not significantly assocxiated with depression. An association with hallucinations and delusions was only observed following three or more moves. Following adjustment for the etiological variables found to be associated with individual categories of psychopathology in model 2, all trends of increasing odds of association with increasing number of moves remained similar to those observed in model 1. Attenuation of odds ratios were generally small following adjustments. However, an association between a history of suicide attempts and a single move was no longer significant.

**Table 4:** Associations between number of Moves before 15 years and Psychopathology (*n*=39,531)

Psychopathology	No Moves (Ref.) *N*=19,937 (50.4%)	Moved 1 Time *N*=11,822 (29.9%)			Moved 2 Times *N*=5,344 (13.5%)			Moved 3 Times or more *N*=2,430 (6.2%)		
	N (%)	N (%)	Model 1 OR (95%CI)	Model 2 OR (95%CI)	N (%)	Model 1 OR (95%CI)	Model 2 OR (95%CI)	N (%)	Model 1 OR (95%CI)	Model 2 OR (95%CI)
Depression^a^ *N*=1,221 (3.1%)	531 (2.7)	349 (3.0)	1.12 (0.97-1.29)	1.08 (0.94-1.24)	199 (3.7)	1.41*** (1.20-1.67)	1.25** (1.06-1.48)	142 (5.8)	2.18*** (1.80-2.64)	1.65*** (1.36-2.01)
Suicide Attempt *N*=550 (1.4%)	222 (1.1)	166 (1.4)	1.67* (1.03-1.55)	1.21 (0.99-1.49)	89 (1.7)	1.51*** (1.18-1.94)	1.33* (1.03-1.71)	74 (3.0)	2.79*** (2.13-3.65)	2.08*** (1.58-2.73)
Somatic Disorder *N*=2,426 (6.1%)	1052 (5.3)	752 (6.4)	1.19*** (1.08-1.31)	1.16** (1.05-1.28)	391 (7.3)	1.39*** (1.23-1.56)	1.27*** (1.13-1.44)	231 (9.5)	1.80*** (1.55-2.09)	1.52*** (1.30-1.77)
Obsessive Disorder *N*=1,423 (3.6%)	601 (3.0)	445 (3.8)	1.20** (1.06-1.37)	1.19** (1.05-1.34)	226 (4.2)	1.35*** (1.15-1.58)	1.25** (1.07-1.46)	151 (6.2)	2.02*** (1.68-2.43)	1.68*** (1.39-2.03)
Hallucinations and delusions *N*=292 (0.7%)	119 (0.6)	93 (0.8)	1.27 (0.97-1.67)	1.22 (0.93-1.61)	43 (0.8)	1.29 (0.91-1.83)	1.14 (0.80-1.62)	37 (1.5)	2.42*** (1.67-3.51)	1.88***(1.29-2.75)

Model1 Adjusted for age, sex, ethnic minority, low family income and rural birth

Model2 Adjusted for age, sex, ethnic minority, low family income, rural birth, childhood maltreatment and left behind (> 6 months)

^a^Also adjusted for rural birth, loss of parent (death) for depression

*p< 0·05, **p< 0·01, ***p< 0·001

### Interaction analysis

We finally carried out interaction analyses between experiencing moves (any) during childhood and putative risk factors found to show significant associations with psychopathology after adjusting for age, sex, ethnic minority status and low family income. Significant interactions were found between reporting any childhood moves and being a left behind child for depression (AOR 1.29*, 95% CI 1.01–1.64) and somatic disorder (AOR 1.25*, 95% CI 1.04–1.50).

We next tested for interactions between a continuous score of total number of moves and age at first move on psychopathology. We found significant interactions for depression (AOR 1.18**, 95% CI 1.09–1.27), suicide attempts (AOR 1.14*, 95% CI 1.02–1.27), somatic disorder (AOR 1.07*, 95% CI 1.01–1.14), and hallucinations and delusions (AOR 1.23**, 95% CI 1.06–1.44) with age at first move reversely recoded.

## Discussion

Our findings suggested lasting effects of residential mobility during childhood and early adolescence on psychopathology in adulthood among this sample of Chinese university students. Our findings corespond to other studies that show that the more moves the greater the association with psychopathology [4, 14, 19, 24, 26, 27, 29–32]. All categories of psychopathology we measured were associated with residential mobility during childhood defined by moves of residence. Associations with three or more moves appeared greatest for reporting suicide attempts, followed by hallucinations and delusions, depression, obsessive-compulsive disorder and somatic disorder. We also confirmed that the younger the students were when they first moved, the stronger the associations with all categories of psychopathology in adulthood. These associations with younger age appeared to be increasing with increasing number of moves. In general, moves that occurred later, when 11 years or older, were not associated with adult psychopathology, except for suicide attempts in the case of students who had experienced 3 or more moves.

Additional key findings of the study were that any experience of residential move during childhood that occurred together with being a left-behind child showed multiplicative effects on depression and somatic disorder in adulthood. The number of moves and younger age at first move also showed multiplicative effects on depression and somatic disorder, but also on suicide attempts and hallucinations and delusions.

### Were the associations with psychopathology confounded by demographics and other etiological risk factors?

We identified potential demographic confounders and observed that younger students in their first year at university and those born in rural areas (whose parents were likely to have moved to urban areas in search of work) were more likely to have experienced residential mobility during childhood and adolescence. However, we did not find an association between number of moves and family income at the time of the survey to suggest that repeated movements had resulted in either increased or decreased wealth in their families relative to those who had not moved, except in the case of a single move which was associated with a reduced likelihood of being from a low-income family. Because we do not have additional information, we could not determine if a single move had resulted in increased family wealth because it was associated with a move involving less family disruption. We also found no associations between number of moves and ethnic minority status. The reduced association between rural birth and a single, or two moves, was unexpected. Our findings suggested that rural birth was generally associated with multiple moves in this student sample. Furthermore, we found rural birth associated with depression. Depression has previously been found to be more prevalent in rural areas of China [53, 54]. Persons from rural areas experience a complex series of interrelated stressors, primarily related to poverty and inequalities in access to healthcare, with reduced life-expectancy [55].

A key aim of the study was to exclude the possibility that the associations we observed between residential mobility and psychopathology were confounded by etiological risk factors, including adverse childhood experiences and maltreatment which have previously been found to be more prevalent among children who experience residential mobility [12, 13]. We initially found dose response associations with reporting having been a left-behind child, physical abuse and emotional neglect. Sexual abuse showed a specific association with 3 or more moves. Left-behind children in China experience anxiety and physical symptoms during childhood [56] and college students who were left behind have been shown to experience persistence of these symptoms in adulthood [57]. Adverse childhood experiences of physical and sexual abuse and neglect are known to exert effects in adulthood on depressed mood [58] and also on psychotic symptoms [59]. Nevertheless, following statistical adjustments, we did not find that the associations between residential mobility in childhood and psychopathology were confounded by other etiological risk factors prevalent among those who experinced moves in childhood. The associations between mobility and other etiological factors with psychopathology were therefore independent of each other.

### Limitations

Strengths of the study include large sample size, few refusals, and a range of mental health outcomes representing common mental disorders. These are conditions of greatest concern in a student sample. However, the study had limitations. The cross-sectional nature of the study meant that causal relationships can only be speculated upon. Although there is some evidence that moving residence may have had an effect, it was not conclusive and our cross-sectional method cannot determine the direction of association between residential instability and the additional risk factors we measured.

We did not have specific measures of family dysfunction during childhood to exclude these factors. We also did not have information on externalising disorders. We therefore did not have information on drug and alcohol misuse which would be a key outcome of study in western universities. We also did not interview participants to confirm diagnostic categories and relied on self-report. However, self-report can compare favourably with clinician assessments [60].

Our findings may not generalise to a general population sample of young persons. Students have higher intelligence. We also do not know whether they experienced fewer childhood adverse experiences because they had more protective factors associated with factors such as greater financial support from their family, or whether they experienced more stress during their upbringing associated with their parents accumulating wealth by migration and/or leaving them with other carers in rural areas in the context of rapid urbanisation of China. In addition, variables measuring childhood maltreatment did not consider frequency and severity. Asking these questions retrospectively may have resulted in recall bias. Nevertheless, previous research indicates that retrospective assessment of childhood adversity has good reliability [61].

Sample effects could explain unexpected lack of association between PEs and family history of severe mental disorder which would be observed in representative community samples. Finally, because cross-sectional data cannot establish causal relationships, the relationship between childhood maltreatment/stressful family factors and psychopathology should be investigated in more comprehensive longitudinal studies.

## Conclusion

In summary, we found that residential mobility during childhood was strongly associated with common mental disorders in early adulthood and that these associations were further increased by younger age at first move and being a left-behind child. Disadvantage and maltreatment during childhood were also strongly associated with psychopathology and were more common among those who experienced residential mobility. Nevertheless, we found residential mobility and disadvantage/maltreatment were independent of each other in their associations with psychopathology.

Future research should establish the complex mechanisms whereby residential mobility leads to psychopathology in early adulthood and the extent to which these involve life-events and disrupted attachments or other factors. Previous research has suggested that effects of early trauma and maltreatment on psychopathology are cumulative [62]. Our findings add to the literature by suggesting that residential mobility in a chinese sample may have multiplicative effects when co-associated with younger age at first move and being a left-behind child, but this will require further confirmation.

## Data Availability

The datasets used and/or analysed during the current study are available from the corresponding author on reasonable request.

## Abbreviations

AOR: Adjusted odds ratios
CI: Confidence interval
OCI-R: The Obsessive-Compulsive Inventory-Revised
PEs: Psychotic experiences
PHQ: Patient Health Questionnaire
SCL-90-R: The Symptom Checklist-90-Revised
SD: Standard deviation
